# The Origins of African *Plasmodium vivax*; Insights from Mitochondrial Genome Sequencing

**DOI:** 10.1371/journal.pone.0029137

**Published:** 2011-12-14

**Authors:** Richard Culleton, Cevayir Coban, Fadile Yildiz Zeyrek, Pedro Cravo, Akira Kaneko, Milijaona Randrianarivelojosia, Voahangy Andrianaranjaka, Shigeyuki Kano, Anna Farnert, Ana Paula Arez, Paul M. Sharp, Richard Carter, Kazuyuki Tanabe

**Affiliations:** 1 Laboratory of Malariology, International Research Centre of Infectious Diseases, Research Institute of Microbial Diseases, Osaka University, Osaka, Japan; 2 Malaria Unit, Institute of Tropical Medicine, Nagasaki University, Nagasaki, Japan; 3 Laboratory of Malaria Immunology, Immunology Frontier Research Center, World Premier Institute for Immunology, Osaka University, Osaka, Japan; 4 Department of Microbiology, School of Medicine, Harran University, Sanliurfa, Turkey; 5 Centro de Malária e outras Doenças Tropicais, Unidade de Parasitologia, Instituto de Higiene e Medicina Tropical, Universidade Nova de Lisboa, Lisbon, Portugal; 6 Instituto de Patologia Tropical e Saúde Pública/CAPES/PVE, Goiânia, Brazil; 7 Department of Medicine, Karolinska University Hospital, Solna, Sweden; 8 Institut Pasteur de Madagascar, Unite de Reserche sur le Paludisme, Antananarivo, Madagascar; 9 Research Institute, International Medical Centre of Japan, Tokyo, Japan; 10 Centre for Immunity, Infection and Evolution, University of Edinburgh, Edinburgh, United Kingdom; Museum National d'Histoire Naturelle, France

## Abstract

*Plasmodium vivax*, the second most prevalent of the human malaria parasites, is estimated to affect 75 million people annually. It is very rare, however, in west and central Africa, due to the high prevalence of the Duffy negative phenotype in the human population. Due to its rarity in Africa, previous studies on the phylogeny of world-wide *P. vivax* have suffered from insufficient samples of African parasites. Here we compare the mitochondrial sequence diversity of parasites from Africa with those from other areas of the world, in order to investigate the origin of present-day African *P. vivax*. Mitochondrial genome sequencing revealed relatively little polymorphism within the African population compared to parasites from the rest of the world. This, combined with sequence similarity with parasites from India, suggests that the present day African *P. vivax* population in humans may have been introduced relatively recently from the Indian subcontinent. Haplotype network analysis also raises the possibility that parasites currently found in Africa and South America may be the closest extant relatives of the ancestors of the current world population. Lines of evidence are adduced that this ancestral population may be from an ancient stock of *P. vivax* in Africa.

## Introduction

*Plasmodium vivax*, responsible for many tens of million cases of malaria globally every year, is the second most abundant malaria parasite of humans after *Plasmodium falciparum* [1]. It has the broadest geographic range of the five malaria parasites infective to man, but appears to be almost completely absent in humans from large parts of western and central Africa, where *P. falciparum* is at its most abundant [2]. This situation is attributed to the high prevalence of the Duffy negative blood condition in the local human populations of this area [3].

The Duffy antigen/receptor for chemokines (DARC) is a transmembrane glycoprotein that is present on epithelial cells [4], endothelial cells [5], and erythrocytes. It is utilised by *P. vivax* parasites as a receptor for attachment to the erythrocyte surface. One of the alleles of the gene that encodes the DARC protein, FY*B^null^, carries a single nucleotide mutation which impairs promoter activity by disrupting a binding site for the h-GATA-1 erythroid transcription factor [6]. This results in the loss of DARC expression on erythrocytes, but does not affect expression in epithelial or endothelial cells. Individuals who are homozygous for this allele thus express no DARC protein on the erythrocyte surface and so are completely protected from the erythrocytic cycle of *P. vivax*.

The Duffy negative phenotype occurs in over 95% of the human population indigenous to west and central Africa, but is extremely rare outside of Africa and the Arabian peninsula [7]. It appears that some form of “selective sweep” event (possibly involving two distinct haplotypes) occurred in the African human population which selected for this mutation, as heterozygosity around the FY*B^null^ allele is low compared to elsewhere on the genome [8]. Population genetics analysis revealed that this selection probably occurred between 6,500 and 97,200 years ago (95% confidence interval) [8]. Given the strong association between the parasite and DARC, it has long been proposed that *P. vivax* was the causative agent for the near fixation of the Duffy negative condition in the African population. Other possible explanations include selection by other pathogens (trans-membrane receptors on the surface of blood cells are obvious targets for cell-invasion [9]), or the hitchhiking of the FY*B^null^ allele during selection for a closely linked gene [8].

*P. vivax* is part of a clade of parasites that predominantly infect catarrhine monkeys in South East Asia, and is generally assumed to have become a parasite of humans via a host switch event. It has been estimated that *P. vivax* diverged from *Plasmodium cynomolgi* (possibly its most closely related sister-species) between 1.17 and 1.60 million years ago, and that the most recent common ancestor of all extant *P. vivax* parasites lived around 400,000 years ago [10]; however, it should be noted that neither of these dates necessarily corresponds to the time at which *P. vivax* became parasitic on humans (*i.e.* the time of the host-switch event). This relatedness to parasites infecting monkeys in Southeast Asia, has led many to speculate that *P. vivax* emerged as a human parasite in this area. However, as the range of the cattarhine monkeys may have extended over large parts of Eurasia at the time of the host-switch, we must consider that this event may have occurred anywhere on these continents where host ranges overlapped [11]. When and where its ancestral line arose and how *P. vivax* spread from its point of origin to its present day distribution, are difficult to ascertain, and remains the subject of much speculation and controversy. A analysis by Mu *et al*, based on mitochondrial genome sequences from 176 *P. vivax* isolates from a wide geographic range, was interpreted to suggest that *P. vivax* originated in Southeast Asia and spread westwards through India and into Africa [12]. This interpretation was supported by Cornejo and Escalante [10] who combined these data with a further 105 mitochondrial sequences from Jongwutiwes *et al* [13]. In all three studies, parasites from Southeast Asia were found to be the most polymorphic, followed by those from Melanesia, the Indian sub-continent, Africa and South America respectively. If we assume that the population showing the highest degree of polymorphism is the most ancient, then these data suggest a simple east-to-west invasion by *P. vivax* from a source population in Southeast Asia. However, currently “most ancient” is not the same as “original”; any assumption to this effect overlooks the complexity of what may have happened between the time and location of an “origin”, in the sense of divergence of a line of organisms from a source population, and the possibly later appearance of the “most ancient” extant population of organisms from that line of descent. Both migration and lineage extinction (bottlenecking) can obscure the geographical origin of the ancestral haplotype.

In an attempt to gain further insight into this question, we apply a population genetics analysis to *P. vivax* isolates from continental Africa, Madagascar and Turkey. We have collected 23 isolates of African/Malagasy *P. vivax* from São Tomé (n = 1), Tanzania (n = 1), Angola (n = 1), Rwanda (n = 1), Niger (n = 1) and Madagascar (n = 18), and 10 isolates from Turkey, from which the mitochondrial genome was sequenced. These sequences were used to construct a haplotype network for African *P. vivax*. We also combine these sequence data with previously reported sequences [12], [13] in order to investigate how the present day African *P. vivax* population relates to parasites from the rest of the world.

## Materials and Methods

### Samples from Turkey

A total of 10 blood samples were collected from patients with patent *P. vivax* malaria at several National Malaria Control Centers within Siverek in Sanliurfa province during the peak season (July to December) of 2004, as previously described [14]. Patent infection was diagnosed by staining of the blood samples with 10% Giemsa's solution and examination with standard light microscopy. All samples were collected after informed consent was obtained from patients or their parents. Sampling authorization was obtained from the Turkish Ministry of Health Sanliurfa Bureau, Sanliurfa, Turkey, and ethical approval was obtained from the Research Institute for Microbial Diseases, Osaka University, Osaka, Japan.

### Samples from Madagascar

Twenty Samples were collected from symptomatic malaria patients from seven locations (Ampasimpotsy, Saharevo, Antananarivo, Ankazobe, Taolagnaro, Antananarivo and Sainte Marie), diagnosed with *P. vivax* by microscopy between 1998 and 2005. *P. vivax* was confirmed in 18 of these samples by PCR diagnosis. All samples were collected after written informed consent was obtained from patients or their parents. Administrative authorizations and ethical clearance were provided by the Ministry of Health and National Ethic Committee, Antananarivo, Madagascar. Seven isolates contained dual infections of distinct mitochondrial genotypes, identified by i) a difference in the repeat length of an internal T-repeat microsatellite marker but no SNPs (six samples), or by ii) overlapping peaks at a single nucleotide (one sample), yielding a total of 25 genotypes in 18 isolates. The internal T-repeat microsatellite marker was subsequently removed from all samples prior to phylogenetic analysis.

### Additional African *P. vivax* samples

Samples of African *P. vivax* were acquired from Angola, São Tomé, Rwanda, Niger and Tanzania. The Angolan sample came from an Angolan traveler reporting to the Central Laboratory of Institute of Tropical Medicine and Hygiene of Lisbon in 2003. *Plasmodium vivax* was diagnosed by microscopy and PCR and blood samples were stored in liquid nitrogen before parasite DNA extraction Ethical clearance for the use of this sample was given by the Ethical Committees of Instituto de Higiene e Medicina Tropical, Lisbon, Portugal, according to EU guidance. The São Toméan sample was collected from the Centro Policlínico de Saúde de Água Grande, in the city of São Tomé in 2004, following ethical approval from the Ministry of Health of the Democratic Republic of São Tomé and Príncipe. Rwandan and Nigerian samples were acquired from Japanese travelers reporting to the International Medical Centre Hospital, Tokyo in 2005 and 2006 respectively. Both patients were diagnosed with *P. vivax* by PCR and microscopy, and blood samples were stored at −80°C before parasite DNA extraction was performed using a QIAamp DNA Blood Mini Kit (QIAGEN, Hilden, Germany) following the manufacturer's instructions. The Tanzanian sample was collected from a patient in Nyamisati village diagnosed by microscopy with *P. vivax* and was part of a longitudinal study granted ethical approval from the National Institute for Medical Research, Dar es Salaam, Tanzania. DNA extraction was performed using a QIAamp DNA Blood Mini Kit (QIAGEN, Hilden, Germany) following the manufacturer's instructions.

For all samples, written informed consent was obtained from the sample donors, with the exception of the Japanese traveller's samples collected at the International Medical Centre Hospital, Tokyo, which does not require written informed consent for the use of parasite DNA from patients.

### Mitochondrial genome sequencing

The entire ∼6 kb mitochondrial genome was sequenced from single *P. vivax* samples from Rwanda, São Tomé, Angola, Tanzania and Niger, 18 samples from Madagascar (yielding 25 individual mitochondrial genomes), and 10 samples from Turkey. The mitochondrial genomic DNA was amplified by PCR, with the entire genome being amplified in two separate reactions. 1 µL of extracted DNA solution was added to 25 µL of distilled H_2_0, 1.75 µL of each primer (5 µM, see **Table S1** for primer sequences), 5 µL of 10X LA PCR™ Buffer II, 5 µL of 25 mM MgCl_2_ solution, 8 µL of dNTP mixture (2.5 mM each) and 0.5 µL of TaKaRa LA *Taq*™ (TaKaRa Bio Inc., Japan) in a 50 µL reaction. The following cycling conditions were applied using a GeneAmp® PCR 9700 thermocycler (Applied Biosystems, USA): 94°C for 1 minute, 40 cycles of 94°C for 20 seconds, 58°C for 30 seconds, 72°C for 4 minutes and a final extension step of 72°C for 10 minutes. PCR products were visualised on 1% agarose gels, and purified using QIAquick® PCR purification kit (QIAGEN, Hilden, Germany) according to the manufacturer's instructions, prior to sequencing. Sequencing reactions were carried out in a 5 µL reaction consisting of 1 µL of purified PCR product (at a concentration of 10 fmol/µL), 1 µL of sequencing primer (**Table S1**), 0.5 µL of distilled H_2_0, 1 µL of BigDye® Terminator v3.1 Cycle Sequencing mixture and 0.5 µL of BigDye® Sequencing Buffer (Applied Biosystems, USA). Cycle conditions were; 96°C for 1 minute, 25 cycles of 96°C for 30 seconds, 50°C for 30 seconds and 60°C for 4 minutes. Sequencing reaction products were purified using DyeEx™ spin protocol for dye-terminator removal (QIAGEN, USA), according to the manufacturer's instructions. Purified products were sequenced on a 3100 Genetic Analyzer (Applied Biosystems, USA), and contiguous sequences were constructed using ATGC version 4.01© (Genetyx Corporation, USA). PCR products were sequenced in both directions, and the entire sequencing process was carried out twice for each isolate including the initial PCR. Singleton mutations were verified by a third round of sequencing of independent PCR products. All sequences were deposited in GeNbank, accession numbers JN788737-JN788776. A multi-“T” repeat microsatellite marker spanning nucleotide positions 2734-2747 was removed from all sequences prior to further analysis.

### Calculation of haplotype diversity

The haplotype diversity index (*h*) for the six geographical sub-divisions considered here (Melanesia, East Asia, the Indian sub-continent, Africa, the Middle East and South America) was calculated using the formula *h* = {n/(n−1)} {1−Σp_i_^2^} [15] where p_i_ is the frequency of the i^th^ mitochondrial genome haplotype and n is the number of individuals sampled. The variance of *h* was calculated using the formula *V* = {2/n(n−1)}[2(n−2){Σ p_i_^3^−(Σp_i_^2^)^2^}+Σp_i_^2^−(Σp_i_^2^)^2^] [16].

### Calculation of nucleotide diversity

Nucleotide diversity (π), the average number of nucleotide differences per site, was determined for each geographical region using the Jukes and Cantor model implemented in the DnaSP v5.10 computer software [17]

### Haplotype network construction

The 40 newly determined sequences from continental Africa, Madagascar and Turkey were used to construct a haplotype network using the NETWORK 4.5.0 programme (Fluxus technology Ltd. 2006). Additionally, 320 sequences were used to construct a larger haplotype network in the same way. Isolates were assigned to geographic populations based on the regions proposed by Mu and colleagues [12] (Melanesia, East Asia, the Indian sub-continent, Africa and South America), with the addition of a new population from the “Middle East” (incorporating samples from Iran and Turkey). Out-group probabilities were generated using TCS 1.21 [18], [19], and give an estimate of the relative age of each haplotype, based on their frequencies and the number of connections to other haplotypes within the network.

## Results

### Origin of the present day African *P. vivax* populations

Nine distinct mitochondrial genome haplotypes were present in samples from Turkey, sub-Saharan Africa and Madagascar, as shown in **Table 1**. Haplotype and nucleotide diversities of worldwide *P. vivax* populations are shown in **Table 2**, and give an estimate for the genetic polymorphism of the mitochondrial genome among samples from the six geographical locations considered. East Asia and Melanesia show the highest diversity, in accordance with previous analyses, which could reflect these parasite populations being the most ancient [10], [12]. Isolates from the Turkey and Iran and those from the Indian sub-continent have comparable haplotype diversities (Turkey plus Iran vs. India, P>0.05), significantly lower than those from East Asia (East Asia vs. India, P = 0.03; East Asia vs. Turkey plus Iran, P = 0.05), and lower, but not statistically significantly so, than those from Melanesia, As expected, diversity was relatively low in South America (P<0.05 for all comparisons, except with Africa), which is generally considered to be the location most recently colonised by *P. vivax*. Interestingly, however, isolates from Africa have the lowest haplotype diversity (P<0.05 for all comparisons, except with South America), which may be indicative of a recent appearance (or possibly a population bottleneck) of this population. In previous analyses [10], [12], [13], the limited number of samples from Africa did not allow firm conclusions to be drawn about the evolutionary history of African *P. vivax*. Excluding those samples from northern Africa as well as those of unknown African origin reveals an even lower haplotype diversity of samples from sub-Saharan Africa. Overall, these results may reflect a migration of *P. vivax* from Asia east into Melanesia, and west into Africa via the Indian sub-continent.

**Table 1:** Summary of positions of SNPs in mitochondrial sequences from *P. vivax* isolates from continental Africa, Turkey and Madagascar.

	Nucleotide position									Number of isolates		
Haplotype	1864	2179	2306	2316	2549	4169	5644	5848	5862	Africa	Turkey	Madagascar
**1**	A	T	A	T	T	T	A	A	G	1	0	0
**2**	A	T	A	T	A	T	A	A	A	4	0	21
**3**	A	T	A	A	A	T	A	A	A	0	0	2
**4**	A	T	A	T	A	A	A	A	A	0	0	1
**5**	A	T	A	T	A	T	A	C	A	0	0	1
**6**	A	C	T	T	A	T	A	A	A	0	1	0
**7**	A	T	A	T	A	T	T	A	A	0	3	0
**8**	C	T	A	T	A	T	A	A	A	0	3	0
**9**	A	T	T	T	A	T	A	A	A	0	3	0
*Total*										5	10	25

**Table 2:** Haplotype and nucleotide diversity of worldwide *P. vivax* mitochondrial genomes.

Region of Origin	Number of Samples	Number of Haplotypes	Haplotype Diversity (*h*) (standard error)	NucleotideDiversity (π)
East Asia	115	52	0.97 (<0.01)	7.5×10^−4^
Melanesia	72	39	0.94 (<0.01)	6.2×10^−4^
Indian sub-continent	31	15	0.86 (±0.05)	4.4×10^−4^
Turkey and Iran	12	6	0.86 (±0.06)	4.4×10^−4^
South America	48	9	0.67 (±0.07)	2.0×10^−4^
Africa	42	12	0.53 (±0.09)	1.8×10^−4^
Sub-Saharan Africa^1^	35	7	0.36 (±0.10)	1.4×10^−4^

1 Excluding those samples from Northern Africa, or of unknown African origin.

**Figure 1:**
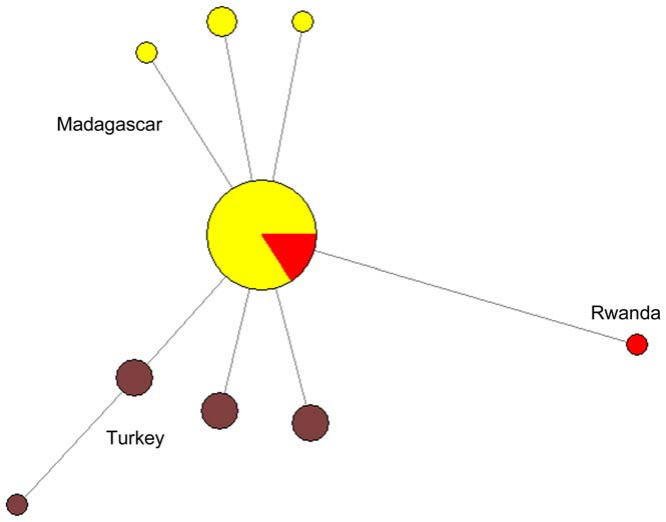
Haplotype network constructed using NETWORK 4.5.0, incorporating 40 *Plasmodium vivax* mitochondrial genome sequences obtained during this work. Node sizes are proportional to haplotype frequency, and branch lengths are indicative of the number of single nucleotide differencesbetween sequences. Node colours indicate the geographic origin of the isolates and are coded as follows: red = continental Africa (n = 5) (Tanzania (1), Angola (1), Niger (1), Rwanda (1), and Sao Tome (1)) yellow = Madagascar (25), brown = Turkey (10). Haplotype diversities (standard error) are, continental Africa, 0.40 (0.24); Madagascar, 0.29 (0.08); Turkey 0.80 (0.08).

A haplotype network was constructed using the mitochondrial genome sequence from the 40 isolates described here (5 from continental Africa, 10 from Turkey and 25 from Madagascar) and is shown in **Figure 1**. The major haplotype found in Madagascar is also found in Tanzania, Sao Tomé, Niger and Angola. A unique haplotype was recorded from the Rwandan isolate. There is a very low haplotype diversity associated with parasites from Madagascar (*h* = 0.29), and continental Africa (*h* = 0.40). The haplotype diversity of the Turkish samples was much higher (*h* = 0.80), with 4 unique haplotypes found among the 10 samples sequenced. Interestingly, haplotypes were not shared between Turkey and Africa/Madagascar.

**Figure 2:**
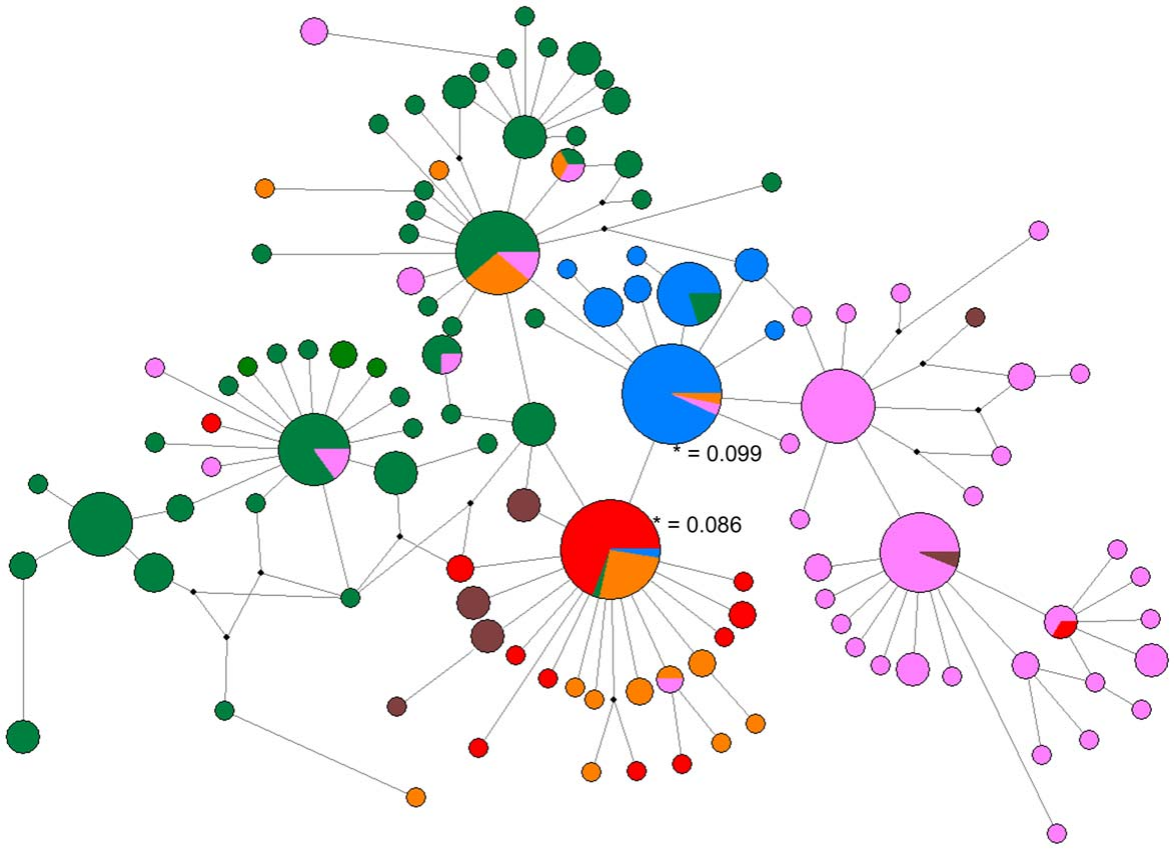
Haplotype network constructed using NETWORK 4.5.0, incorporating 320 *P. vivax* mitochondrial genome sequences (280 previously published [**12]**, [13****], and 40 samples from this work). Node sizes are proportional to haplotype frequency. Node colours indicate the geographic origin of the isolates and are coded as follows; red = Africa, blue = South America, green = Asia, pink = Melanesia, orange = the Indian sub-continent, brown = Middle East (Turkey and Iran). Hypothetical intermediates are indicated by small black points. The haplotypes marked with an asterisk are the two with the highest out-group probabilities (see **Table S2**). Haplotype diversities for geographical regions are given in **Table 2**.

These 40 sequences were also combined with 280 previously published mitochondrial genomes [12], [13] in order to construct a new haplotype network comprised of *P. vivax* parasites from a worldwide distribution (**Figure 2**). There were 117 distinct haplotypes among the 320 sequences considered, with 99 polymorphic nucleotide positions. The network follows the same general pattern as previously published trees [10], [12], with large numbers of private haplotypes from East Asia and Melanesia. There is more haplotype diversity among *P. vivax* isolates than is seen with *P. falciparum* [20], with larger numbers of private haplotypes in all areas. Although haplotypes segregate according to geographical location, there are many types that are shared between locations. There are relatively few private haplotypes from Africa/Madagascar and South America, which is reflected in their low haplotype numbers (**Table 2**). Samples from Turkey cluster with those from Africa/Madagascar and India, whereas the two samples reported to be from Iran [13] group with those found in Melanesia. The very large number of private haplotypes originating in East Asia and Melanesia suggests that sampling in these areas is below that required for saturation, and further collection from these areas may reveal additional new haplotypes.

Estimates of out-group probabilities (which are indicative of which haplotypes are most likely to be ancestral, **Table S2**), reveal two haplotypes with out-group weights of over 0.08 (marked with asterisks in **Figure 2**). The most likely ancestral haplotype from our analysis (out-group weight, 0.099) is present in India and Melanesia and South America (where it is the dominant haplotype). The second most likely ancestral haplotype (out-group weight, 0.086) is found in Africa/Madagascar, South America, India and Asia.

## Discussion

### On the origins of extant populations of P. vivax

The haplotype network constructed from the mitochondrial genome of worldwide *P. vivax* isolates reflects the genetic diversity of global extant *P. vivax* populations. While the network reveals a similar picture to that previously presented by Mu *et al* [12], and Cornejo and Escalante [10], inclusion of a further 30 samples from sub-Saharan Africa and Madagascar has increased the probability of reaching sampling saturation. This increases our confidence that the low haplotype diversity seen in the latter population is not a sampling effect.

The Melanesian and East Asian populations show the greatest haplotype and nucleotide diversities. This could suggest that these populations are the most ancient, and that they are ancestral to *P. vivax* samples found elsewhere. Indeed, the way in which haplotype diversity decreases from East Asia to the Indian sub-continent to Africa/Madagascar is consistent with the westward movement of present day *P. vivax* from a population somewhere in East Asia. Africa/Madagascar has the lowest haplotype diversity among all worldwide populations, lower even than South America, which is generally considered to be the last continent to have been colonised by *P. vivax*. Thus our results appear to show that the *present day* African/Madagascan *P. vivax* in humans may be relatively young compared to other populations.

Other possibilities remain, however. Regardless of the geographic location where *P. vivax* the species originated, an event that probably took place many hundreds of thousands of years ago, numerous complex geographic movements of *P. vivax* stocks have almost certainly taken place since. For example there is no obvious reason why *P. vivax* should not have been endemic across Africa prior to the emergence of Duffy negativity in human populations on this continent less than one hundred thousand years ago [8]. Such a stock of *P. vivax* would have been driven close to extinction under the near fixation of the Duffy negative mutation in the indigenous human host population. Subsequently the parasites would have been re-introduced into Africa from an external *P. vivax* stock when human migration patterns became favourable to the reintroduction of both the Duffy positive trait and of *P. vivax* itself.

One way in which this could have happened is via the import of East Asian/Indian *P. vivax* to the coastal areas of east Africa by early sea-going traders, in much the same way as it is thought that *P. falciparum* was introduced to South America [21]. The fact that the same parasite mitochondrial haplotypes are seen in India and in east Africa and Madagascar, but that haplotype diversity is greater in India is consistent with this idea. The absence of shared haplotypes between the Middle East and Africa, is also consistent with a re-introduction of *P. vivax* via a sea route across the Indian Ocean and not by land through the Middle East.

Several aspects of the haplotype network warrant further discussion. First, the out-group probability analysis identified the haplotype most commonly found in South America, haplotype number 2 (h2), as the most likely ancestral type. The haplotype identified as the second most likely ancestral type is that found most commonly in India and Africa (h40), which differs from h2 by a single nucleotide substitution. The central position of h2 within the network, located intermediate between clusters of isolates from (on the one side) Melanesia and (on the other side) East Asia, seems quite anomalous if the South American parasite population is the most recently derived. We therefore propose an alternative hypothesis regarding the history of *P. vivax* that is consistent with the h2 haplotype being the most ancient.

Under this hypothesis current South American *P. vivax* is derived from the now extinct European *P. vivax* which is itself derived from an ancient *P. vivax* stock, found in its time only in Africa. From this ancient African stock all extant *P. vivax* is derived spreading first to Europe and later to the Indian sub-continent and on to East Asia; independent lineages spread from Africa to Melanesia. Subsequent to the dispersion of this *P. vivax* stock out of Africa, the Duffy negative condition appeared and spread in the African human population leading to the near extinction of *P. vivax* in humans in Africa itself. In the post-Columbus era the European/ancient African stock of *P. vivax* crossed the Atlantic to the Americas, where it survives to this day only in South America, *P. vivax* in Europe and North America having been driven to extinction by the late 20^th^ century. In this scenario the greater genetic diversity among *P. vivax* haplotypes seen in the East Asian and Melanesian populations may be partly due to the larger pools of parasites in these regions compared to those in South America, Africa and the Indian subcontinent where bottlenecking of *P. vivax* stocks may have occurred during their introductions.

The paradigm of the absolute dependence of *P. vivax* on DARC for red blood cell invasion has recently been challenged by a number of studies that describe infections in Duffy negative individuals. In 2006, Ryan and colleagues demonstrated the presence of *P. vivax* parasites in mosquitoes living in a region populated almost exclusively by Duffy negative individuals in western Kenya, and some evidence for blood-stage parasites in a number of Duffy negative children from the same region [23]. The following year, Cavasini and colleagues reported the detection of *P. vivax* DNA from Duffy negative individuals in Brazil [22]. Following these observations, a landmark report from Madagascar was published in 2010, claiming that *P. vivax* parasites frequently infected Duffy negative people on that island [24]. This finding raises two main possibilities; firstly, the *P. vivax* parasites endemic to this region may have adapted to become able to invade Duffy negative red blood cells. This would be a very serious situation, given the proximity of Madagascar to sub-Saharan Africa, and the potential of such a parasite to spread within the Duffy negative populations of that continent. Secondly, the possibility exists that the “Duffy negative” individuals infected with the parasite may be of a previously undescribed Duffy phenotype that allows the invasion of *P. vivax* parasites, while maintaining some character of Duffy negativity. Our results show that, at least for those samples collected from the 7 regions of Madagascar described between 1998 and 2005 and analysed in this study, the vast majority of the parasites shared identical mitochondrial genome sequences with parasites also found in continental Africa, suggesting that movement of parasites between Madagascar and Africa has occurred previously, although we cannot be sure of the direction of the transfer.

We have previously shown that despite very high levels of Duffy negativity in the local human populations, *P. vivax* does occur in some regions of West central Africa [14], although it is scarce [25]. How transmission is maintained in regions where 95–100% of the population are supposedly resistant to *P. vivax*, may appear to be enigmatic. However, as we have previously pointed out, at a sufficiently high vectorial capacity (and in west and central Africa the vectorial capacities for human malaria are typically amongst the highest in the world) transmission of a malaria parasite can be readily sustained where only 1–5% of the human population are susceptible to it [14]. Altogether, therefore, the evidence supports the likely presence of *P. vivax*, albeit at low levels, in humans in west and central Africa.

Intriguingly, very recent reports show that chimpanzees and gorillas from several locations across Central Africa harbour parasites with mitochondrial DNA sequences almost identical to *P. vivax* [26], [27]. There is, therefore, an animal pool of parasites very similar, or identical, to *P. vivax* in the heart of this region from which *P. vivax* has, until recently, appeared to be entirely absent. These observations raise questions concerning the movements of *P. vivax*-like parasites through historical and prehistorical times in Africa. Further samples of *P. vivax*-like parasites isolated from non-human primates in Africa could shed light on these questions.

## Supporting Information

Table S1 **Primers used for mitochondrial genome sequencing.**
(DOCX)

Table S2 **Outgroup weights for haplotypes (>0.01).**
(DOCX)

## References

[1] Carter R, Mendis KN (2002). Evolutionary and historical aspects of the burden of malaria.. Clin Microbiol Rev.

[2] Guerra CA, Snow RW, Hay SI (2006). Mapping the global extent of malaria in 2005.. Trends Parasitol.

[3] Miller LH, Mason SJ, Clyde DF, McGinniss MH (1976). The resistance factor to Plasmodium vivax in blacks. The Duffy-blood-group genotype, FyFy.. N Engl J Med.

[4] Horuk R, Martin AW, Wang Z, Schweitzer L, Gerassimides A (1997). Expression of chemokine receptors by subsets of neurons in the central nervous system.. J Immunol.

[5] Hadley TJ, Lu ZH, Wasniowska K, Martin AW, Peiper SC (1994). Postcapillary venule endothelial cells in kidney express a multispecific chemokine receptor that is structurally and functionally identical to the erythroid isoform, which is the Duffy blood group antigen.. J Clin Invest.

[6] Tournamille C, Colin Y, Cartron JP, Le Van Kim C (1995). Disruption of a GATA motif in the Duffy gene promoter abolishes erythroid gene expression in Duffy-negative individuals.. Nat Genet.

[7] Cavalli-Sforza LL (1994). Africa..

[8] Hamblin MT, Di Rienzo A (2000). Detection of the signature of natural selection in humans: evidence from the Duffy blood group locus.. Am J Hum Genet.

[9] Pease JE, Murphy PM (1998). Microbial corruption of the chemokine system: an expanding paradigm.. Semin Immunol.

[10] Cornejo OE, Escalante AA (2006). The origin and age of Plasmodium vivax.. Trends Parasitol.

[11] Carter R (2003). Speculations on the origins of Plasmodium vivax malaria.. Trends Parasitol.

[12] Mu J, Joy DA, Duan J, Huang Y, Carlton J (2005). Host switch leads to emergence of Plasmodium vivax malaria in humans.. Mol Biol Evol.

[13] Jongwutiwes S, Putaporntip C, Iwasaki T, Ferreira MU, Kanbara H (2005). Mitochondrial genome sequences support ancient population expansion in Plasmodium vivax.. Mol Biol Evol.

[14] Culleton R, Ndounga M, Zeyrek F, Coban C, Casimiro P (2009). Evidence for the transmission of Plasmodium vivax in the Republic of the Congo, West Central Africa.. J Infect Dis.

[15] Nei M (1987). Molecular Evolutionary Genetics.

[16] Sakihama N, Ohmae H, Bakote'e B, Kawabata M, Hirayama K (2006). Limited allelic diversity of Plasmodium falciparum merozoite surface protein 1 gene from populations in the Solomon Islands.. Am J Trop Med Hyg.

[17] Librado P, Rozas J (2009). DnaSP v5: a software for comprehensive analysis of DNA polymorphism data.. Bioinformatics.

[18] Castelloe J, Templeton AR (1994). Root probabilities for intraspecific gene trees under neutral coalescent theory.. Mol Phylogenet Evol.

[19] Clement M, Posada D, Crandall KA (2000). TCS: a computer program to estimate gene genealogies.. Mol Ecol.

[20] Joy DA, Feng X, Mu J, Furuya T, Chotivanich K (2003). Early origin and recent expansion of Plasmodium falciparum.. Science.

[21] Anderson TJ, Haubold B, Williams JT, Estrada-Franco JG, Richardson L (2000). Microsatellite markers reveal a spectrum of population structures in the malaria parasite Plasmodium falciparum.. Mol Biol Evol.

[22] Cavasini CE, Mattos LC, Couto AA, Bonini-Domingos CR, Valencia SH (2007). Plasmodium vivax infection among Duffy antigen-negative individuals from the Brazilian Amazon region: an exception?. Trans R Soc Trop Med Hyg.

[23] Ryan JR, Stoute JA, Amon J, Dunton RF, Mtalib R (2006). Evidence for transmission of Plasmodium vivax among a duffy antigen negative population in Western Kenya.. Am J Trop Med Hyg.

[24] Menard D, Barnadas C, Bouchier C, Henry-Halldin C, Gray LR (2010). Plasmodium vivax clinical malaria is commonly observed in Duffy-negative Malagasy people.. Proc Natl Acad Sci U S A.

[25] Culleton R, Mita T, Ndounga M, Unger H, Cravo P (2008). Failure to detect Plasmodium vivax in West and Central Africa by PCR species typing.. Malar J.

[26] Krief S, Escalante AA, Pacheco MA, Mugisha L, Andre C (2010). On the diversity of malaria parasites in African apes and the origin of Plasmodium falciparum from Bonobos.. PLoS Pathog.

[27] Liu W, Li Y, Learn GH, Rudicell RS, Robertson JD (2010). Origin of the human malaria parasite Plasmodium falciparum in gorillas.. Nature.

