# Supplementary material for: The Origins of African Plasmodium vivax; Insights from Mitochondrial Genome Sequencing
Source: PLoS One. 2011 Dec 14;6(12):e29137. doi: 10.1371/journal.pone.0029137 (PMC3237592; doi:10.1371/journal.pone.0029137)
Supplement: Table S1 — Primers used for mitochondrial genome sequencing. (DOCX) [file pone.0029137.s001.docx]

**Supplementary Table1**

**Table S1** Primers used for mitochondrial genome sequencing

| Primer name | Sequence (5’- 3’) |
| --- | --- |
| *Forward* |  |
| PvmtF505 | CTATTCATAGAGACAACTAATGGCA |
| PvmtF1001 | CATGCAGGACGGAGATTACCCGA |
| PvmtF1539 | CATACATAAATAAAACGGTAGATAGGGA |
| PvmtF2027 | ATAACCATACAATTTCAACAAAATGCCA |
| PvmtF2479 | GGAGATAAACTAAAATGTAAAATACCCCA |
| PvmtF4046 | ATCTATTTTGGTTTTTTGGACATCCTGA |
| PvmtF4635 | GCATTTTCTTGGATTTAATGTAATGCCT |
| PvmtF4978 | AGAGAATTATGGAGTGGATGGTGT |
| PvmtF5488 | GGTATAATTCCATTATCTCATCCAGA |
| PvmtF3576 | AATATAATGCCAGGATTATTTGGAGGA |
| PvmtF0 | AAGTTAGGATAATGAAATTATAGTTACCA |
| PvmtF2959 | TACTAAGATAAAGAACTCCAGGCGT |
| *Reverse* |  |
| PvmtR619 | CAACAGGAGATTATATTTTGGTAGTGGA |
| PvmtR1169 | AGAAGTTATATTCTGGAAGCGTCTGT |
| PvmtR1638 | TTCCATTGGAATGAGAGTTCACCGT |
| PvmtR2143 | TGTATGATACTAATAGTGAATGGTCA |
| PvmtR2583 | TTCTATATTTTCATCATTAGTATCAGGA |
| PvmtR3441 | AACAGATAATAAAAAACCATAACTACCA |
| PvmtR3931 | GCATTACAGATGTAATTATTAATGACCA |
| PvmtR4433 | GCTGCATTACCTAATATTACTCCTGT |
| PvmtR4933 | TATAACGACTTGCTAAAAATACACCTGT |
| PvmtR5431 | GACTTAATAGATTTGGATAGAAGGGT |
| PvmtR0 | TTAACATAATTATAACCTTACGGTCTGT |
| PvmtR3088 | CAACATAACATTTTTTAGTCCCATGCT |
