# Supplementary material for: The Origins of African Plasmodium vivax; Insights from Mitochondrial Genome Sequencing
Source: PLoS One. 2011 Dec 14;6(12):e29137. doi: 10.1371/journal.pone.0029137 (PMC3237592; doi:10.1371/journal.pone.0029137)
Supplement: Table S2 — Outgroup weights for haplotypes (>0.01). (DOCX) [file pone.0029137.s002.docx]

**Table S2** Outgroup weights for haplotypes (>0.01)

| Haplotype Number | Outgroup weight^1^ | Geographic distribution |
| --- | --- | --- |
| 2 | 0.099 | South America / India / Melanesia |
| 40 | 0.086 | Africa / India / South America / Asia |
| 46 | 0.060 | Asia |
| 27 | 0.058 | Asia / Melanesia / India |
| 7 | 0.054 | Melanesia |
| 77 | 0.043 | Turkey |
| 41 | 0.040 | India |
| 35 | 0.039 | South America |
| 14 | 0.039 | Melanesia / Middle East |
| 69 | 0.038 | Africa |
| 79 | 0.038 | Middle East |
| 82 | 0.031 | Asia |
| 99 | 0.029 | South America |
| 19 | 0.029 | Asia / Melanesia |
| 63 | 0.029 | South America |
| 4 | 0.022 | Africa and Melanesia |
| 50 | 0.022 | Asia |
| 88 | 0.021 | Melanesia / India / Asia |
| 29 | 0.018 | Melanesia |
| 47 | 0.016 | Asia |
| 103 | 0.016 | Asia |
| 53 | 0.016 | Asia |
| 25 | 0.016 | Melanesia |
| 85 | 0.012 | Asia |
| 93 | 0.012 | Asia |
| 80 | 0.012 | Asia |

^1^ determined using TCs1.21 (Clement, Posada and Crandall, molecular ecology 9 (10) 2000)
